# Supplementary material for: Assessment of standard HIV testing services delivery to injured persons seeking emergency care in Nairobi, Kenya: A prospective observational study
Source: PLOS Glob Public Health. 2022 Oct 14;2(10):e0000526. doi: 10.1371/journal.pgph.0000526 (PMC10021732; doi:10.1371/journal.pgph.0000526)
Supplement: S1 Table — (DOCX) [file pgph.0000526.s001.docx]

**Supplements:**

**S1 Table. Likert Items and Topical Focus**

| **Likert Item** | **Topical Focus** |
| --- | --- |
| All patients in the emergency department should be offered ways to undergo HIV testing even if they did not come seeking HIV testing. | Testing should be offered for all patients |
| Patients in the emergency department specifically with injuries should be offered ways to undergo HIV testing even though they are there for evaluation and treatments for their injuries. | Testing should be offered for injured patients specifically |
| The room and physical space for standard provider-initiated testing and counselling for HIV is adequate to provide patient comfort with testing in the emergency department | The physical space and room are acceptable for testing |
| In the emergency department maintaining confidentiality or privacy for standard provider-initiated testing and counselling for HIV is adequate. | Confidentiality and trust are sufficient for testing |
| The relationship and trust created between the patient and healthcare provider in the emergency department is adequate for standard provider-initiated testing and counselling for HIV. | Patient-provider relationships are adequate for testing |
| The time a patient has in the emergency department is enough for standard provider-initiated testing and counselling for HIV. | The treatment time a patient has is enough for testing |
