# Supplementary material for: Assessment of standard HIV testing services delivery to injured persons seeking emergency care in Nairobi, Kenya: A prospective observational study
Source: PLOS Glob Public Health. 2022 Oct 14;2(10):e0000526. doi: 10.1371/journal.pgph.0000526 (PMC10021732; doi:10.1371/journal.pgph.0000526)
Supplement: S2 Table — (DOCX) [file pgph.0000526.s002.docx]

**Supplements:**

**S2 Table.** **Characteristics Stratified by Facilities-Based Offering of HIV Test Services**

|  | **HIV Testing Offered**  **(n=63)** | **HIV testing Not Offered**  **(n=583)** |  |
| --- | --- | --- | --- |
| **Variable** | **n (%) or Median (IQR)** | **n (%) or Median (IQR)** | **P value** |
| *Sex* |  |  |  |
| Male | 50(80.4%) | 517 (88.7%) | 0.032 |
| Female | 13 (10.6%) | 66 (11.3%) |  |
| *Age (years)* |  |  |  |
| 18-25yrs | 17 (27.0%) | 178 (30.5%) | 0.311 |
| 26-44yrs | 42 (66.7%) | 345 (58.0%) |  |
| > 44yrs | 4 (6.4%) | 68 (11.4%) |  |
| *Relationship Status* |  |  |  |
| Single | 21 (33.3%) | 196 (33.8%) | 0.001 |
| Married | 27 (42.9%) | 274 (47.2%) |  |
| In a relationship | 8 (12.7%) | 69 (11.9%) |  |
| Divorced / Widowed | 4 (6.4%) | 39 (6.7%) |  |
| Wishes not to disclose | 3 (4.8%) | 1 (0.2%) |  |
| Missing / Unknown | 0 (0.0%) | 1 (0.2%) |  |
| *Education* |  |  |  |
| Completed primary schooling (or less) | 29 (46.0%) | 223 (38.3%) | 0.037 |
| Completed secondary schooling | 29 (46.0%) | 234 (40.1%) |  |
| Greater than Secondary schooling | 5 (8.0%) | 126 (21.6%) |  |
| *Has Primary Care Provider* |  |  |  |
| No | 28 (44.4%) | 320 (55.0%) | 0.003 |
| Yes | 34 (54.0%) | 263 (45.1%) |  |
| Missing / Unknown | 1(1.6%) | 0 (0.0%) |  |
| *Uses Recreational Alcohol* |  |  |  |
| Yes | 34 (54.0%) | 280 (48.6%) | 0.621 |
| No | 28 (45.2%) | 295 (51.2%) |  |
| Missing / Unknown | 1 (1.6%) | 1 (0.2%) |  |
| *Uses Recreational Substances (other than alcohol) ^*^* |  |  |  |
| Yes | 8 (12.7%) | 133 (22.8%) | 0.065 |
| No | 55 (87.3%) | 450(77.6%) |  |
| *Timing of Last HIV Testing^#^* |  |  |  |
| < 6 months prior | 29 (46.0%) | 236 (40.5%) | 0.395 |
| > 6 months prior | 34 (54.0%) | 347 (59.5%) |  |
| *Frequency of Condom use during intercourse* |  |  |  |
| Always | 7 (11.1%) | 107 (18.4%) | 0.339 |
| Sometimes | 15 (23.8%) | 127 (21.8%) |  |
| Never | 32 (50.8%) | 298 (51.1%) |  |
| Wishes not to disclose | 3 (4.8%) | 11 (1.9%) |  |
| Missing | 6 (9.5%) | 40 (6.9%) |  |
| *Number of Body regions injured* | 2 (1-2) | 1 (1-2) | 0.206 |
| *Transferred from Another Health Facility* |  |  |  |
| Yes | 22 (34.9% | 250 (42.9%) | 0.416 |
| No | 41 (65.1%) | 331 (56.8%) |  |
| Missing | 0 (0.0%) | 2 (0.3%) |  |
| Emergency Department Arrival Time |  |  |  |
| Day (6am – 6pm) | 41 (65.1%) | 318 (54.6%) | 0.110 |
| Night (6pm – 6 am) | 22 (34.9%) | 265 (45.5%) |  |
| *Emergency Department Disposition* |  |  |  |
| Discharge | 28 (44.4%) | 333 (57.2%) | 0.478 |
| Admitted to Inpatient Service | 32 (50.8%) | 226 (38.8%) |  |
| Admitted to Operating Theater | 2 (3.2%) | 10 (1.7%) |  |
| Transferred to another facility | 1 (1.6%) | 5 (0.9%) |  |
| Left Prior to Care Completion | 0 (0.0%) | 7 (1.3%) |  |
| Deceased | 0 (0.0%) | 1 (0.2%) |  |

* Included substances were classified as depressant, psychogenic or stimulant
