# Supplementary material for: Assessment of standard HIV testing services delivery to injured persons seeking emergency care in Nairobi, Kenya: A prospective observational study
Source: PLOS Glob Public Health. 2022 Oct 14;2(10):e0000526. doi: 10.1371/journal.pgph.0000526 (PMC10021732; doi:10.1371/journal.pgph.0000526)
Supplement: Attachment — Submitted filename: PLOS_Respons_letter.pdf [file pgph.0000526.s004.pdf]

5 September, 2022

Dear Editor,

We appreciate the chance to amend and resubmit our manuscript, "**Assessment of Standard HIV Testing Services Delivery to Injured Persons Seeking Emergency Care in Nairobi, Kenya: A Prospective Observational Study**" to *PLOS Global Public Health* for consideration for publication as an original article. Please note that the manuscript has been changed to address the points raised by the editor and peer reviewers. Each point is outlined below with the corresponding response and associated alterations in the manuscript explained.

As previously noted, we believe this manuscript will be important in informing discourse and future research to support the advancement of HIV programming from emergency care settings, and as such is well suited for publication in *PLOS Global Public Health*, a new and key journal with appropriate scope for this important topic.

We appreciate the ongoing consideration of our revised manuscript for publication and for giving us the opportunity to improve on the work based on the constructive feedback. Please contact us with any questions regarding this submission. We look forward to your future correspondence.

Comments & Authors' Responses:

Editor/Journal:

Please include a complete copy of PLOS' questionnaire on inclusivity in global research in your revised manuscript.

- **Authors' Response:** The form has been completed and uploaded with the revision

Please amend your online detailed Financial Disclosure statement. This is published with the article. It must therefore be completed in full sentences and contain the exact wording you wish to be published.

a. State the initials, alongside each funding source, of each author to receive each grant.

b. State what role the funders took in the study. If the funders had no role in your study, please state: "The funders had no role in study design, data collection and analysis, decision to publish, or preparation of the manuscript."

- **Authors' Response:** The financial disclosure statement was updated as directed.

Please update your online Competing Interests statement. If you have no competing interests to declare, please state: "The authors have declared that no competing interests exist."

- **Authors' Response:** The competing interest statement has been amended as directed.

We have noticed that you have uploaded Supporting Information files, but you have not included a list of legends. Please add a full list of legends for your Supporting Information files after the references list.

- **Authors' Response:** The list of legends has been added to the revision as requested.

We do not publish any copyright or trademark symbols that usually accompany proprietary names, eg (R), (C), or TM (e.g. next to drug or reagent names). Please remove all instances of trademark/copyright symbols throughout the text, including ™ on page 5.

- **Authors' Response:** The text has been removed.

Please review your reference list to ensure that it is complete and correct. If you have cited papers that have been retracted, please include the rationale for doing so in the manuscript text, or remove these references and replace them with relevant current references. Any changes to the reference list should be mentioned in the rebuttal letter that accompanies your revised manuscript. If you need to cite a retracted article, indicate the article's retracted status in the References list and also include a citation and full reference for the retraction notice.

- **Authors' Response:** The references have been reviewed and are correct for the manuscript. If there are any needed changes, please let us know.

Could you maybe include whether this project was part of a larger prospective study? I.e., did the participants give informed consent to a study about HTS in the emergency department, or to a larger study on additional outcomes? I can imagine if it were the former, those consenting might be more inclined to accept HTS when offered (but conversely it would seem odd that HTS was offered so infrequently by the staff if they knew a study evaluating this in their unit was ongoing). Therefore it makes more sense that these outcomes were embedded in a larger project looking at emergency care in general. This would be useful information to include somewhere.

- **Authors' Response:** The study is part of a larger body of work (grant K23AI145411) however the specific study reported on does represent an independent piece of research with the *a priori* aims as described in the submission pertaining to HTS. The clinical staff (who were independent of the research personnel) were aware of the study being undertaken but by design were not influenced in any way or directed to alter practice. As well, the enrolled participants were aware that the work was evaluating HTS in the emergency department which could have impacted acceptance of testing if offered. It is important to note however that the informed consent process ensured that the participants understood that HIV testing was not being provided by the study and that exposure to HTS would be based on the stand care provided during the emergency treatment course. To highlight this important point text was added to the methods and discussion sections of the manuscript.

Could you maybe speculate why the offering of HTS was so low?

- **Authors' Response:** The authors agree this is an important consideration and text was added to the discussion around this point. Of note, a follow up qualitative study to evaluate barriers and opportunities for HTS will be completed soon in the same study setting this year to obtain an in-depth assessment of this.

Reviewer 1:

To understand better the contextual situation at this particular one study site it would be important to know how alcohol use prior to presentation at ED may or may not have influenced consent and testing procedures. More than half of the study population admitted to social drinking, majority of reasons for attending ED were accidents and falls which is kmown to have alcoho; as a contributing factor. Exclusion to study population were not detailed enough to understand better if consiousness was seriously affected by trauma or alcohol or emergency conditions to fullfill eligibility criteria.

- **Authors' Response:** The reviewer raises an excellent point about the available data and the methods were clarified around this. Specifically, any screened patient with altered mental status due to any reason (including intoxication) was excluded. As no serologic markers was available the ability to discern between altered mental status secondary to intoxication versus injury could not be done and as such that level of fidelity can not be provided in the results. This is an acknowledged limitation which has been identified in the discussion and although it does impact the generalizability it would not alter the internal validity of the results found for the enrolled population.

Large majority of study population knew already their serostatus, almost half from a recent test less than 6 months ago which is high as compared to other urban areas in the region which may affect consent and could be elaborated on in the discussion

- **Authors' Response:** The informed consent procedures did make the participants aware that the study was designed to evaluate ED-based HTS but also explicitly explained that testing services were not required with enrollment and would occur based on the in place clinical approaches independent of the study. Given this it is felt to be less likely that this would have impacted the consent and enrollment processes. These details and important clarification were added to the manuscript.

It was not that clear from the data presentation if during the 2-6 hours of stay at the ED, most or all had given consent or that consent was provided later during admission for those half requiring admission.

- **Authors' Response:** All enrollment was completed in the ED as close to time of arrival as possible without impeding care. This was clarified in the methods section.

The conclusion to expand PITC during ED should take into account a point earlier made in the paper that the HIV service provision at KNH is exceptional and 24 hrs present and a call for making available those services with trained staff and 24hrs easily accessible supplies to be made stronger.

- **Authors' Response:** The authors agree that the resource access does support the feasibility of improved ED-based HTS and this was added to the discussion section in a tempered manner to be cognizant of the tone and implications of drawing such a conclusion without being able to fully account for all systems factors external to the research.

What were the main reasons that 308 of the 563 patients excluded could not be consented? Were they all critically ill? Language barriers?

- **Authors' Response:** As described in the prior response the 308 patients unable to provide consent were due to altered mental status but categorization of the etiology of this could not be done. All study personnel were native to the location and fluent in the required languages of the population.

Reviewer 2:
It is possible that the 308 patients unable to be consented for this study would also not be able to be consented for HTS in the ED. If so, this study reflects a pragmatic number of patients that could be consented for HTS if the systems were in place in the ED.

- **Authors' Response:** The authors agree with this assessment by the reviewer and this point is provided in the discussion highlighting the potential impacts on the generalizability.

Was there any data/knowledge regarding how clinicians or others determine to offer HTS to patients? Is it only clinical staff (Medical officers) that offer and order HTS, or are there separate health care workers that offer this at KNH

- **Authors' Response:** The VCT personnel are tasked with general screening of patients without and formal criteria or mandate beyond the provision of universal access which was one of the key reasons the study was undertaken to better understand to what extent this penetrates to the high-risk subpopulation of injured persons. Beyond the VCT personnel screening, clinical providers can refer patients for ED-based HTS at their discretion. This information was added to the methods section to provider a more comprehensive understanding of the clinical context.

I found just two typo/grammatical errors: In line 303, please remove the word "towards". In line 317, please change the vernacular term, "missingness" to "missing data" or something else.

- **Authors' Response:** Thank you for the identification of these they have been corrected.

Most Sincerely,

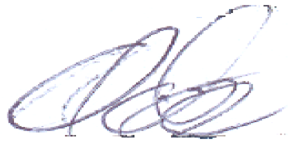

Adam R. Aluisio MD, MSc, DTM&H
Warren Alpert School of Medicine, Brown University
Department of Emergency Medicine
